# Vaccine Development against the Renin-Angiotensin System for the Treatment of Hypertension

**DOI:** 10.1155/2019/9218531

**Published:** 2019-08-14

**Authors:** Tatsuhiko Azegami, Hiroshi Itoh

**Affiliations:** ^1^Health Center, Keio University, 4-1-1 Hiyoshi, Kohoku-ku, Yokohama-shi, Kanagawa 223-8521, Japan; ^2^Department of Internal Medicine, School of Medicine, Keio University, 35 Shinanomachi, Shinjuku-ku, Tokyo 160-8582, Japan

## Abstract

Hypertension is a global public health issue and the most important preventable cause of cardiovascular diseases. Despite the clinical availability of many antihypertensive drugs, many hypertensive patients have poor medication adherence and blood pressure control due, at least partially, to the asymptomatic and chronic characteristics of hypertension. Immunotherapeutic approaches have the potential to improve medication adherence in hypertension because they induce prolonged therapeutic effects and need a low frequency of administration. The first attempts to reduce blood pressure by using vaccines targeting the renin-angiotensin system were made more than half a century ago; however, at the time, a poor understanding of immunology and the mechanisms of hypertension and a lack of optimal vaccine technologies such as suitable antigen design, proper adjuvants, and effective antigen delivery systems meant that attempts to develop antihypertensive vaccines failed. Recent advances in immunology and vaccinology have provided potential therapeutic immunologic approaches to treat not only infectious diseases but also cancers and other noncommunicable diseases. One important biotechnology that has had a major impact on modern vaccinology is virus-like particle technology, which can efficiently deliver vaccine antigens without the need for artificial adjuvants. A human clinical trial that indicated the effectiveness and safety of a virus-like particle-based antiangiotensin II vaccine marked a turning point in the field of therapeutic antihypertensive vaccines. Here, we review the history of the development of immunotherapies for the treatment of hypertension and discuss the current perspectives in the field.

## 1. Introduction

Hypertension is a global public health issue and the most important preventable cause of cardiovascular disease worldwide [1, 2]. Annually, cardiovascular death accounts for approximately 17 million deaths, of which hypertension is responsible for 45% of deaths due to heart disease and 51% of deaths due to stroke worldwide [3]. The global medical costs associated with hypertension are estimated at US$370 billion per year [4], suggesting that hypertension imposes a tremendous economic burden on both individual patients and healthcare systems. Therefore, the control of hypertension is extremely important not only for the prevention of life-threatening complications but also as part of cost-containment strategies in global healthcare.

In the treatment of hypertension, the lack of clinical symptoms is thought to result in poor medication adherence and the cessation of therapy at the patients' own judgment [5]. Poor medication adherence is the main cause of failure to achieve adequate blood pressure control and the development of uncontrolled hypertension [6]. Therefore, new strategies to control hypertension that effectively decrease blood pressure and also improve medication adherence are required.

Immunotherapeutic approaches for the treatment of hypertension, which are often referred to as “hypertension vaccines,” have the potential to improve health outcomes, reduce healthcare costs, and increase medication adherence because they induce prolonged therapeutic effects and have a low frequency of administration [7]. Traditionally, vaccines are used for the prevention of infectious diseases and are usually derived from live attenuated or inactivated microorganisms. In contrast, essential hypertension is a multifactorial disease arising from the combined action of genetic, environmental, and other unknown factors, and there is little evidence of the direct involvement of specific microorganisms. Therefore, endogenous pressor substances, such as those that constitute the renin-angiotensin system (RAS), are the main targets for the development of a hypertension vaccine.

In 1898, Tigerstedt and Bergmann first reported that intravenous injection of extract from rabbit kidney into other rabbits induced an increase in blood pressure [8]. Although this finding was a landmark discovery in the pathogenesis of hypertension, it went unregarded for the next few decades. It was not until it was revealed that renal ischemia induced an increase in blood pressure in dogs [9] and numerous investigators tried to elucidate the pathogenesis of hypertension using renal ischemia models that renin received extensive attention, which resulted angiotensin (Ang) being identified as a substance that causes renal hypertension [10, 11].

Although the first half of the 20th century produced many new insights into the pathogenesis of hypertension, especially with respect to the RAS, the development of treatments for hypertension was lacking. Early experiments targeted renin for the treatment of hypertension, but few chemical compounds were known to directly and strongly inhibit the function of renin [12]. Therefore, prompted by the successful development of vaccines for infectious diseases, active and passive immunotherapies targeting renin were examined as early approaches for the treatment of hypertension.

**Figure 1:**
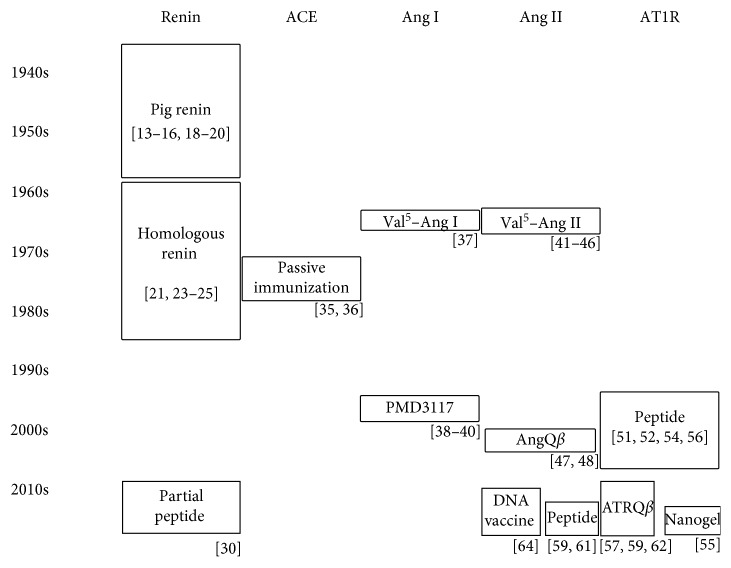
Historical development of hypertension vaccines. ACE: angiotensin converting enzyme; Ang: angiotensin; AT1R: angiotensin II type 1 receptor.

Here, we review the history of the development of immunotherapies for the treatment of hypertension (Figure 1) and discuss the current perspectives in the field.

## 2. Renin Vaccines

In the 1940s, the first attempts were made to develop an immunotherapy to control hypertension. Page et al. succeeded in reducing blood pressure in hypertensive humans, dogs, and rats by subcutaneously or intramuscularly injecting extracts of pig kidney [13]. In their human study, the amount of kidney extract administered daily to hypertensive patients for several weeks was equivalent to 800 to 1000 g of whole fresh kidney [13]. The injection of renal extract did not cause an immediate fall in blood pressure, but after several days, a clear reduction in blood pressure was observed. However, using such a large amount of kidneys per patient is not practical in the clinic, and therefore, another method is required. In terms of safety, there were no serious adverse events directly related to the therapy; however, local skin reactions at the injection site and low-grade fever were observed [13]. Around the same time, another group also demonstrated that daily intramuscular injection of pig renin for two months decreased blood pressure in hypertensive dogs, from an average femoral blood pressure of 164 mmHg to 114 mmHg, without any adverse events [14]. These findings showed the antihypertensive properties of heterologous renin (i.e., renin vaccine) and marked a great advance in the treatment of hypertension.

In a subsequent clinical study, vaccination of hypertensive patients with pig renin twice a week for several weeks or months induced the production of anti-renin antibodies in some vaccines but did not induce a reduction in blood pressure [15]. This failure to attenuate hypertension in humans may have been a result of the insufficient neutralization potency by human antibodies against pig renin because other studies indicated that antibodies against pig renin have little effect on human renin [16, 17]. Subsequent studies confirmed the antihypertensive effects of immunization with pig kidney extract in dogs [16, 18, 19] and monkeys [20], but not in humans [16]. Later, it was found that homologous renin could be chemically altered to make it antigenic; in dogs with renal hypertension, subcutaneous administration of acetylated dog renin three times a week for 10 weeks was shown to progressively reduce blood pressure to the normotensive level [21]. Therefore, examinations began into using homologous renin for immunization of human hypertensive patients.

Adjuvants were also used to amplify the immunogenicity of homologous renin. Freund's complete adjuvant (FCA) and Freund's incomplete adjuvant (FIA) are commonly used adjuvants in animal research. FIA is essentially paraffin oil containing mannide monooleate as a surfactant, and FCA is FIA with the addition of heat-killed mycobacterium [22]. When a vaccine antigen is mixed with these adjuvants, a viscous water-in-oil emulsion is formed that is suitable for injection and stimulates the innate immunity. Subcutaneous injection of purified human renin together with FCA at the first administration and FIA at following administrations induced a dramatic reduction in blood pressure in normotensive marmosets [23]. Interestingly, these antihypertensive effects were induced after only three immunizations at intervals of three weeks [23], whereas most previous renin vaccines needed more than three injections per week for several weeks or even months before an effect was seen. However, despite this success, all of the vaccinated marmosets unexpectedly died from autoimmune renal disease [23]. Reexaminations were immediately performed in which marmosets were immunized with recombinant human renin in combination with Freund's adjuvants, and rats were immunized with mouse renin extracted from the submandibular gland of mice in combination with Freund's adjuvants [24, 25]. From these studies, it was concluded that vaccination against renin induced autoimmune interstitial disease localized in the kidney but not the heart, aorta, or other organs [24, 25]. This autoimmune interstitial nephritis was characterized by the presence of immunoglobulins colocalized with renin, interstitial periarteriolar cellular infiltration, and fibrosis around the juxtaglomerular apparatus, suggesting both humoral and cellular immune responses at the major sites of renin production, storage, and release [24, 25]. Similarly, vaccine-induced autoimmune diseases were also reported in studies evaluating an amyloid beta-peptide 42 vaccine (AN1792) for Alzheimer's disease. In a phase II study in patients with Alzheimer's disease, 6% of patients intramuscularly immunized with AN1792 developed meningoencephalitis independent of antibody titer [26]. A Th1-based T-cell response induced by AN1792 may explain this induction of autoimmune meningoencephalitis [27].

Because the pathogenesis of the autoimmune diseases caused by immunization with renin or AN1792 could not be fully elucidated, the development of renin vaccines was stalled for a long time. However, recently, the use of partial renin peptides has been examined for the development of a safe and effective hypertension vaccine. The active site of human renin is a deep cleft between the N- and the C-terminal domains [28]. Human renin possesses a flap segment that lies across the cleft and holds the substrate at the catalytic site [29]. Recently, a potential epitope of rat and human renin, which included a catalytic site or flap sequence, was antigenic and hydrophobic and had low or no similarity with other host proteins was reported [30]. Six short peptides comprising amino acid sequences located within residues 32–38, 72–81, or 215–221 of the N-terminal of human or rat renin (named hR32, hR72, hR215, rR32, rR72, and rR215) were produced and coupled to keyhole limpet hemocyanin (KLH) [30]. Conjugation of a peptide epitope to a carrier protein is often used to overcome immune tolerance against self-antigens. KLH is a widely used carrier protein that also contains a T-cell epitope, meaning that KLH conjugates can induce specific immune responses to small molecular mass haptens [31]. Subcutaneous immunization with these partial renin peptides coupled to KLH together with Freund's adjuvants-elicited antigen-specific antibodies in rats [30]. Of the six peptides examined, rR32 is the most promising because its subcutaneous administration to spontaneously hypertensive rats (SHRs) produced the strongest reduction in plasma renin activity and blood pressure without immune-mediated damage [30]. Thus, epitope-based vaccines could be novel lead molecules for the development of renin vaccines, but further studies are needed to clarify their efficacy and safety.

## 3. Immunotherapies against Angiotensin-Converting Enzyme

Skeggs and colleagues first isolated angiotensin-converting enzyme (ACE; formerly hypertensin-converting enzyme) in 1956 [32]. In the 1970s, research to examine the localization of ACE began in which normotensive animals were immunized with purified heterologous ACE to obtain fluorescein-labeled anti-ACE antibodies [33, 34]. Around the same time, an animal experiment evaluating passive immunization with anti-ACE antibody revealed that passive immunization with goat antibodies specific for rabbit ACE suppressed the vasopressor response to angiotensin I [35]. However, anti-rabbit ACE antibodies intravenously injected to rabbits were found to bind to the alveolar capillary wall, leading to lethal pulmonary edema [36]. As a result of safety concerns about anti-ACE antibody-induced lethal lung damage, development of an ACE vaccine was stopped.

## 4. Angiotensin I Vaccines

In 1970, Johnston et al. first evaluated the effect of an asparaginyl-5-valine (Val^5^) Ang I vaccine (with Freund's adjuvants) on blood pressure in bilateral-kidney-wrap rabbits but reported that this vaccine did not suppress the pressor response to kidney wrapping [37]. Three decades later, Gardiner et al. created a series of Ang I vaccines comprising Ang I peptide coupled to tetanus toxoid (TT), diphtheria toxin, or KLH carrier proteins and mixed with diethylaminoethyl cellulose or aluminum hydroxide adjuvants, and they were able to report the first antihypertensive effects of Ang I vaccines [38]. Of the combinations examined, vaccination with 5 *μ*g of Ang I-TT mixed with aluminum hydroxide at 0, 14, and 28 days induced the highest anti-Ang I antibody titer and suppressed Ang I-induced pressor responses in Sprague Dawley (SD) rats [38]. In a subsequent study, they compared the effectiveness of using TT and KLH as carrier proteins for immunization in humans; although Ang I-TT and Ang I-KLH induced equivalent IgG antibody responses and attenuation of Ang I-induced pressor responses in SD rats, only Ang I-KLH induced IgG antibodies in humans [39]. Epitopic suppression resulting from previous exposure to TT is a plausible reason for the poor antibody response in humans. In addition, contrary to expectation, the anti-Ang I IgG response generated by the Ang I-KLH vaccine was insufficient to attenuate pressor responses following Ang I or Ang II challenge in human healthy volunteers [39]. In a subsequent randomized double-blind placebo-controlled phase II clinical trial, the antihypertensive effect of three or four subcutaneous immunizations with PMD3117 vaccine (100 *μ*g of Ang I-KLH adsorbed on an aluminum hydroxide adjuvant) was evaluated in patients with essential hypertension; it was found that immunization with PMD3117 increased plasma renin and decreased urinary aldosterone, suggesting blockade of the RAS, but failed to decrease blood pressure [40].

## 5. Angiotensin II Vaccines

In 1968, Oken and Biber reported the antihypertensive effects of an Ang II vaccine: immunization with Val^5^-Ang II coupled to rat albumin together with FCA three or four times at intervals of at least two weeks did not reduce blood pressure but did suppress vasopressor responses against the administration of exogenous Ang II in SD rats [41]. Subsequent examination of the Val^5^-Ang II vaccine revealed that it also had suppressive effects on Ang II-induced vasopressor responses in renovascular hypertensive rabbits [42] and that it reduced blood pressure in renovascular hypertensive rats [43]. However, other studies did not show any preventive and therapeutic effects of Ang II vaccine on hypertension in renovascular hypertensive rabbits [37, 44], renovascular hypertensive rats [37, 45], or SHRs [46].

Virus-like particles (VLPs) are a new immunological approach for the induction of B-cell responses that has been applied to the development of a hypertension vaccine [47, 48]. Because VLPs are formed by structural viral proteins that are able to self-assemble and have antigenic epitopes that induce humoral immune responses, incorporation of target antigens into VLPs can improve the presentation of foreign antigens to the immune system [49]. In fact, immunization with Ang II peptide conjugated to VLP derived from the RNA of bacteriophage Q*β* (AngQ*β*) reduced blood pressure (systolic blood pressure reduced by up to 21 mmHg) without serious adverse events in SHRs [47]. Thereafter, in a phase I clinical study, a single injection with 100 *μ*g of AngQ*β* to healthy volunteers elicited anti-Ang II IgG antibodies with no serious adverse events, but most participants showed local adverse events such as erythema, edema, pain, and induration at the injection site [47]. In 2008, human phase IIa testing of AngQ*β* in hypertensive patients (AngQ*β* study 1) was conducted and successfully showed that AngQ*β* has antihypertensive effects [48]. In study 1, 72 patients with mild-to-moderate hypertension were randomly assigned to receive three subcutaneous injections of 100 or 300 *μ*g of AngQ*β*, or placebo, at 0, 4, and 12 weeks. The titer of IgG antibodies against Ang II was strongly increased after the second injection in vaccinated subjects, and the half-life of antibody titer after the final immunization was 17 weeks. In the participants given 300 *μ*g of AngQ*β*, the mean ambulatory blood pressure at 2 weeks after the final vaccination was significantly reduced by 9.0/4.0 mmHg compared with the placebo. In particular, morning blood pressure was markedly lowered by 25/13 mmHg, suggesting that AngQ*β* has particularly strong efficacy against the morning surge in blood pressure.

Next, a phase II study of AngQ*β* testing an accelerated treatment regimen with injection of 300 *μ*g of AngQ*β* at 0, 2, 4, 6, and 10 weeks was conducted (AngQ*β* study 2). Although the antibody titers induced by this intensive regimen were 5 times those observed in study 1, the reduction in blood pressure (−2.5/−0.9 mmHg) was much lower [50]. In an additional study testing a higher dose of AngQ*β* administered following the accelerated treatment regimen (540 *μ*g at 0, 2, 4, 6, and 10 weeks; AngQ*β* study 3), antibody titers were also higher than those observed in study 1 but the reduction in blood pressure was much lower and did not achieve statistical significance compared with placebo. Contrary to expectations, antibody affinity (i.e., strength of binding to Ang II) was lower in studies 2 and 3 than in study 1 [50]. One possible reason for this was that affinity maturation was impaired by too frequent antigen administration. Following these results, the pharmaceutical development of AngQ*β* was discontinued.

## 6. Angiotensin II Type 1 Receptor Vaccines

In 1999, Zelezna et al. demonstrated that immunization against angiotensin II type 1 receptor (AT1R) attenuated the development of hypertension in young SHRs [51]. Their AT1R vaccine was composed of bovine gamma globulin-conjugated AT1R partial peptides corresponding to residues 14–23 of the N-terminal part of AT1 receptor together with Freund's adjuvant. Five immunizations with AT1R vaccine from 1 month of age at one-month intervals induced antigen-specific IgG antibodies and reduced mean arterial pressure by about 10% [51]. However, immunization with the AT1R vaccine did not change blood pressure in mature hypertensive SHRs or normotensive Wistar Kyoto rats. Another AT1R vaccine (residues 165–191), created by Wang et al., also did not decrease blood pressure in normotensive Wistar rats [52].

Because the second extracellular loop, especially residue Phe^182^, of AT1R is an important part of the Ang II binding pocket [53], we and another group examined the second extracellular loop of AT1R as a vaccine target [54–57]. Subcutaneous immunization with AT1R peptide (residues 181–187) conjugated to TT or KLH in combination with Freund's adjuvants successfully attenuated hypertension in SHRs [54, 56]. Single immunization with AT1R-KLH induced antigen-specific serum IgG antibodies, but the antibody titer was lower than those produced by three or six immunizations, and it did not decrease blood pressure in SHRs [54]. In contrast, three doses of AT1R-KLH vaccine induced an effective immune response and antihypertensive effect equivalent to those provided by six doses of vaccine [54]. Three doses of subcutaneous immunization with 100 *μ*g of AT1R-KLH in combination with FCA at first administration and FIA at following administrations to SHRs at 4, 6, and 8 weeks of age attenuated the elevation of blood pressure (systolic blood pressure of vaccinated rats −44 mmHg vs. control rats); this attenuation of hypertension was equivalent to that provided by continuous administration of candesartan cilexetil (0.1 mg·kg^−1^·day^−1^) and was sustained for 25 weeks after the final immunization [54].

VLPs derived from bacteriophage Q*β* were also used to develop a vaccine targeting AT1R [57]. Chen et al. covalently conjugated AT1R peptide (residues 181–187) to VLP Q*β* and immunized SHRs with 100 *μ*g of AT1R peptide conjugated to VLP Q*β* (ATRQ*β*-001) in combination with aluminum hydroxide at 6 and 8 weeks of age. ATRQ*β*-001 vaccination decreased systolic blood pressure by 19 mmHg and attenuated left ventricular hypertrophy [57]. The antihypertensive effect generated by ATRQ*β*-001 was sustained for 84 days after the final immunization.

## 7. Organ-Protective Effects of Vaccines

Recently accumulated evidence has clearly indicated that vaccination targeting the RAS induces not only a reduction in blood pressure but also attenuates hypertensive organ damage, including that in the kidney, heart, brain, and arteries. Previously, we reported that three doses of subcutaneous vaccination with 100 *μ*g of AT1R-KLH in combination with Freund's adjuvant prevented hypertensive kidney damage caused by NG-nitro-L-arginine methyl ester- (L-NAME-) induced endothelial injury in SHRs at a level equivalent to continuous administration of candesartan cilexetil, whereas no renoprotective effect against L-NAME-induced kidney damage was observed in hydralazine-treated SHRs that had blood pressure controlled to a level almost equal to that of the AT1R-KLH-vaccinated SHRs, indicating that ATR1-KLH vaccination prevents hypertensive kidney damage independent, at least partially, of the vaccine's antihypertensive effects [54]. Subsequently, Ding et al. reported a protective effect of AT1R vaccine on diabetic nephropathy: three subcutaneous immunizations with 400 *μ*g of ATRQ*β*-001 in combination with aluminum hydroxide reduced blood pressure and concomitantly prevented podocyte injury and renal fibrosis and inflammation, resulting in decreases in urine protein and serum creatinine levels in streptozotocin-induced diabetic SD rats [58].

The cardioprotective effects of Ang II vaccine and AT1R vaccine have also been reported. Three subcutaneous immunizations (5 *μ*g/dose) of Ang II-KLH-conjugate vaccine in combination with Freund's adjuvants prevented cardiac dysfunction and attenuated cardiac fibrosis after myocardial infarction in SD rats [59]. Similarly, ATRQ*β*-001, when subcutaneously injected twice before and three times after the induction of myocardial infarction, attenuated myocardial inflammation and fibrosis in C57BL/6 mice, resulting in improvement of cardiac function and survival [60].

In addition to cardioprotective effects, Ang II-KLH vaccine also been shown to induce cerebroprotective effects [61]. Three doses of subcutaneous immunization with Ang II-KLH vaccine together with Freund's adjuvants induced anti-Ang II serum IgG antibody that penetrated into ischemic lesions across a broken blood-brain barrier, resulting in inhibition of oxidative stress and reduction in infarction size after induction of cerebral infarction in Wistar rats [61]. Vaccination against AT1R may also prevent atherosclerosis preceding the onset of organ damage; in apolipoprotein E-null mice, multiple subcutaneous injections of ATRQ*β*-001 vaccine reduced plaque size in the aortic sinus and suppressed macrophage accumulation in the plaque [62].

## 8. Current and Future Perspectives

In addition to VLPs, other recent advances in vaccine technologies (i.e., DNA vaccination and the use of nanoparticles for vaccine delivery) have also been used in hypertension vaccine development. DNA vaccines have several advantages over traditional vaccines, including prolonged antigen expression, improved stability, and more rapid production [63]. Koriyama et al. created a plasmid vector encoding a fusion protein between Ang II and hepatitis B core (HBc) as an Ang II DNA vaccine [64]. Three intradermal immunizations to SHRs with 100 *μ*g of DNA without exogenous adjuvant by using a needleless injector at two-week intervals successfully induced antigen-specific antibody responses that were sustained for at least 6 months and promoted a decrease in systolic blood pressure that was correlated with antibody titer and sustained for at least 6 months [64]. Further analyses revealed that the Ang II-HBc fusion protein contained immunogenic T-cell epitopes for the activation of T cells that meant that exogenous adjuvants were not needed, suggesting that the Ang II DNA vaccine induced sufficient humoral immune responses to suppress Ang II function while avoiding the activation of unnecessary self-reactive T cells [64]. Following these successful results, Ang II DNA vaccine is being evaluated in an ongoing phase I/II clinical study.

Our group created a nasal AT1R vaccine using a nanoparticle-based vaccine delivery system [7]. All previously reported anti-RAS vaccines were administered by systemic injection via the subcutaneous, intradermal, or intramuscular routes, but it has been reported from a clinical trial that injection of Ang II vaccine often induced localized skin adverse reactions such as edema, induration, and physiological pain at the injection site [48]. The mucosal route, including the oral and nasal routes of administration, is a noninvasive vaccine strategy to avoid such injection-related problems. However, because intranasal administration of peptide-based antigen alone often fails to induce a sufficient antigen-specific immune response due to the presence of physiological defense mechanisms in the digestive and respiratory tracts, an efficient antigen-delivery system is required [65]. We used a nanometer-sized hydrogel (nanogel) made from cationic cholesteryl group-bearing pullulan (cCHP) that effectively and safely delivers antigens to antigen-presenting dendritic cells in the nasal epithelium [65]. AT1R-PspA antigen comprising AT1R partial peptides (residues 181–187) coupled to pneumococcal surface protein A (PspA) was incorporated into the cCHP nanogel together with cyclic di-GMP adjuvant. Five doses of intranasal administration of AT1-PspA vaccine containing 10 *μ*g of vaccine antigen at one-week intervals induced the production of AT1R-specific serum IgG antibody and attenuated the elevation of systolic blood pressure (−16.8 mmHg vs. control) in SHRs [7]. Interestingly, intranasal immunization with AT1R-PspA vaccine also has the potential to protect from lethal pneumococcal infection because AT1R-PspA antigen also possesses epitopes of *Streptococcus pneumoniae* [7]. The concept of simultaneous protection against two distinct common human disorders, in this case, a communicable disease and a noncommunicable disease, may be an innovative and creative approach for the future development of hypertension vaccines because hypertension is a risk factor and poor prognostic factor for infectious diseases.

## 9. Conclusions

Evidence accumulated over more than half a century regarding the efficacy of therapeutic hypertension vaccines indicates that vaccines targeting the RAS can decrease blood pressure and attenuate hypertensive organ damage. The immunotherapeutic approach has advantages over traditional antihypertensive agents in that it can improve medication adherence; this is because it induces prolonged therapeutic effects and so needs a low frequency of administration. Vaccines against the RAS offer a promising means of reducing the economic burden caused by hypertension and its related disorders while increasing the rate of patients with adequate blood pressure control. Although no vaccine product is clinically approved as a therapeutic vaccine against hypertension, Ang II DNA vaccine is currently under clinical investigation (phase I/II trial) and expected to be proven its safety and effectiveness. In the near future, the immunotherapeutic approach may result in the development of novel hypertension therapies.

## References

[1] Yusuf S., Hawken S., Ôunpuu S. (2004). Effect of potentially modifiable risk factors associated with myocardial infarction in 52 countries (the INTERHEART study): case-control study. *The Lancet*.

[2] O’Donnell M. J., Chin S. L., Rangarajan S. (2016). Global and regional effects of potentially modifiable risk factors associated with acute stroke in 32 countries (INTERSTROKE): a case-control study. *The Lancet*.

[3] (April 2019). https://apps.who.int/iris/bitstream/handle/10665/79059/WHO_DCO_WHD_2013.2_eng.pdf.

[4] Frieden T. R., Jaffe M. G. (2018). Saving 100 million lives by improving global treatment of hypertension and reducing cardiovascular disease risk factors. *The Journal of Clinical Hypertension*.

[5] Burnier M. (2006). Medication adherence and persistence as the cornerstone of effective antihypertensive therapy. *American Journal of Hypertension*.

[6] Macedo A. F., Morgado M., Castelo-Branco M., Rolo S., Pereira L. (2010). Predictors of uncontrolled hypertension and antihypertensive medication nonadherence. *Journal of Cardiovascular Disease Research*.

[7] Azegami T., Yuki Y., Nakahashi R., Itoh H., Kiyono H. (2018). Nanogel-based nasal vaccines for infectious and lifestyle-related diseases. *Molecular Immunology*.

[8] Kirthana Kunikullaya U., Ananthakrishnan V., Goturu J. (2012). Robert Tigerstedt and the discovery of renin—a revisit. *International Journal of Cardiology*.

[9] Goldblatt H., Lynch J., Hanzal R. F., Summerville W. W. (1934). Studies on experimental hypertension: I. The production of persistent elevation of systolic blood pressure by means of renal ischemia. *Journal of Experimental Medicine*.

[10] Braun-Menendez E., Fasciolo J. C., Leloir L. F., Muñoz J. M. (1940). The substance causing renal hypertension. *The Journal of Physiology*.

[11] Page I. H., Helmer O. M. (1940). A crystalline pressor substance (angiotonin) resulting from the reaction between renin and renin-activator. *Journal of Experimental Medicine*.

[12] Kokubu T., Ueda E., Fujimoto S. (1968). Peptide inhibitors of the renin-angiotensin system. *Nature*.

[13] Page I. H., Helmer O. M., Kohlstaedt K. G., Fouts P. J., Kempf G. F. (1941). Reduction of arterial blood pressure of hypertensive patients and animals with extracts of kidneys. *Journal of Experimental Medicine*.

[14] Wakerlin G. E., Johnson C. A., Gomberg B., Goldberg M. L. (1941). Reduction in the blood pressures of renal hypertensive dogs with hog renin. *Science*.

[15] Goldblatt H., Haas E., Lamfrom H. (1951). Antirenin in man and animals. *Transactions of the Association of American Physicians*.

[16] Helmer O. M. (1958). Studies on renin antibodies. *Circulation*.

[17] Lamfrom H., Haas E., Goldblatt H. (1954). Studies on antirenin. *American Journal of Physiology-Legacy Content*.

[18] Katz J. I., Skom J. H., Wakerlin G. E., Graham L., Keith B., Speer R. (1957). Pathogenesis of spontaneous and pyelonephritic hypertension in the dog. *Circulation Research*.

[19] Wakerlin G. E. (1958). Antibodies to renin as proof of the pathogenesis of sustained renal hypertension. *Circulation*.

[20] Frank M. H. (1963). Renin in experimental renal hypertension in monkeys. *Circulation Research*.

[21] Deodhar S. D., Haas E., Goldblatt H. (1964). Production of antirenin to homologous renin and its effect on experimental renal hypertension. *Journal of Experimental Medicine*.

[22] Billiau A., Matthys P. (2001). Modes of action of Freund’s adjuvants in experimental models of autoimmune diseases. *Journal of Leukocyte Biology*.

[23] Michel J. B., Guettier C., Philippe M., Galen F. X., Corvol P., Menard J. (1987). Active immunization against renin in normotensive marmoset. *Proceedings of the National Academy of Sciences of the United States of America*.

[24] Michel J.-B., Huang H., Guettier C. (1989). Renin immunization and angiotensin converting enzyme inhibition in the normotensive marmoset. *Journal of Hypertension Supplement: Official Journal of the International Society of Hypertension*.

[25] Michel J. B., Sayah S., Guettier C. (1990). Physiological and immunopathological consequences of active immunization of spontaneously hypertensive and normotensive rats against murine renin. *Circulation*.

[26] Orgogozo J.-M., Gilman S., Dartigues J.-F. (2003). Subacute meningoencephalitis in a subset of patients with AD after A*β*42 immunization. *Neurology*.

[27] Pride M., Seubert P., Grundman M., Hagen M., Eldridge J., Black R. S. (2008). Progress in the active immunotherapeutic approach to Alzheimer’s disease: clinical investigations into AN1792-associated meningoencephalitis. *Neurodegenerative Diseases*.

[28] Rahuel J., Priestle J. P., Grutter M. G. (1991). The crystal structures of recombinant glycosylated human renin alone and in complex with a transition state analog inhibitor. *Journal of Structural Biology*.

[29] Blundell T., Sibanda B. L., Pearl L. (1983). Three-dimensional structure, specificity and catalytic mechanism of renin. *Nature*.

[30] Qiu Z., Chen X., Zhou Y. (2013). Therapeutic vaccines against human and rat renin in spontaneously hypertensive rats. *PLoS One*.

[31] Harris J. R., Markl J. (1999). Keyhole limpet hemocyanin (KLH): a biomedical review. *Micron*.

[32] Skeggs L. T., Kahn J. R., Shumway N. P. (1956). The preparation and function of the hypertensin-converting enzyme. *Journal of Experimental Medicine*.

[33] Caldwell P., Seegal B., Hsu K., Das M., Soffer R. (1976). Angiotensin-converting enzyme: vascular endothelial localization. *Science*.

[34] Silverstein E., Pertschuk L. P., Friedland J. (1979). Immunofluorescent localization of angiotensin converting enzyme in epithelioid and giant cells of sarcoidosis granulomas. *Proceedings of the National Academy of Sciences of the United States of America*.

[35] Caldwell P. R., Wigger H. J. (1976). Angiotensin-converting enzyme: effect of antienzyme antibody in vivo. *FEBS Letters*.

[36] Barba L. M., Caldwell P. R., Downie G. H., Camussi G., Brentjens J. R., Andres G. (1983). Lung injury mediated by antibodies to endothelium. I. In the rabbit a repeated interaction of heterologous anti-angiotensin-converting enzyme antibodies with alveolar endothelium results in resistance to immune injury through antigenic modulation. *Journal of Experimental Medicine*.

[37] Johnston C. I., Hutchinson J. S., Mendelsohn F. A. (1970). Biological significance of renin angiotensin immunization. *Circulation Research*.

[38] Gardiner S. M., Auton T. R., Downham M. R. (2000). Active immunization with angiotensin I peptide analogue vaccines selectively reduces the pressor effects of exogenous angiotensin I in conscious rats. *British Journal of Pharmacology*.

[39] Downham M. R., Auton T. R., Rosul A. (2003). Evaluation of two carrier protein-angiotensin I conjugate vaccines to assess their future potential to control high blood pressure (hypertension) in man. *British Journal of Clinical Pharmacology*.

[40] Brown M. J., Coltart J., Gunewardena K., Ritter J. M., Auton T. R., Glover J. F. (2004). Randomized double-blind placebo-controlled study of an angiotensin immunotherapeutic vaccine (PMD3117) in hypertensive subjects. *Clinical Science*.

[41] Oken D., Biber T. (1968). Biologically effective immunization against angiotensin. *American Journal of Physiology-Legacy Content*.

[42] Eide I., Aars H. (1969). Renal hypertension in rabbits immunized with angiotensin. *Nature*.

[43] Christlieb A. R., Biber T. U. L., Hickler R. B. (1969). Studies on the role of angiotensin in experimental renovascular hypertension: an immunologic approach. *Journal of Clinical Investigation*.

[44] Louis W. J., Renzini V., Macdonald G. J., Boyd G. W., Peart W. S. (1970). Renal-clip hypertension in rabbits immunised against angiotensin II. *The Lancet*.

[45] Eide I. (1972). Renovascular hypertension in rats immunized with angiotensin II. *Circulation Research*.

[46] Christleib A. R., Hickler R. B. (1972). Blood pressure response and antibody formation in spontaneously hypertensive rats and normal albino rats after immunization against angiotensin II. *Endocrinology*.

[47] Ambühl P. M., Tissot A. C., Fulurija A. (2007). A vaccine for hypertension based on virus-like particles: preclinical efficacy and phase I safety and immunogenicity. *Journal of Hypertension*.

[48] Tissot A. C., Maurer P., Nussberger J. (2008). Effect of immunisation against angiotensin II with CYT006-AngQb on ambulatory blood pressure: a double-blind, randomised, placebo-controlled phase IIa study. *The Lancet*.

[49] Grgacic E. V. L., Anderson D. A. (2006). Virus-like particles: passport to immune recognition. *Methods*.

[50] (March 2019). http://kuros.ch/uploads/news/id127/Cytos_Press_E_091110.pdf.

[51] Zelezna B., Veselsky L., Velek J., Dobesova Z., Zicha J., Kunes J. (1999). Influence of active immunization against angiotensin AT1 or AT2 receptor on hypertension development in young and adult SHR. *Physiological Research*.

[52] Wang B., Liao Y.-H., Zhou Z. (2005). Arterial structural changes in rats immunized by AT1-receptor peptide. *Heart and Vessels*.

[53] Baleanu-Gogonea C., Karnik S. (2006). Model of the whole rat AT1 receptor and the ligand-binding site. *Journal of Molecular Modeling*.

[54] Azegami T., Sasamura H., Hayashi K., Itoh H. (2012). Vaccination against the angiotensin type 1 receptor for the prevention of L-NAME-induced nephropathy. *Hypertension Research: Official Journal of the Japanese Society of Hypertension*.

[55] Azegami T., Yuki Y., Hayashi K. (2018). Intranasal vaccination against angiotensin II type 1 receptor and pneumococcal surface protein A attenuates hypertension and pneumococcal infection in rodents. *Journal of Hypertension*.

[56] Zhu F., Liao Y. H., Li L. D. (2006). Target organ protection from a novel angiotensin II receptor (AT1) vaccine ATR12181 in spontaneously hypertensive rats. *Cellular & Molecular Immunology*.

[57] Chen X., Qiu Z., Yang S. (2013). Effectiveness and safety of a therapeutic vaccine against angiotensin II receptor type 1 in hypertensive animals. *Hypertension*.

[58] Ding D., Du Y., Qiu Z. (2016). Vaccination against type 1 angiotensin receptor prevents streptozotocin-induced diabetic nephropathy. *Journal of Molecular Medicine*.

[59] Watanabe R., Suzuki J. I., Wakayama K. (2017). A peptide vaccine targeting angiotensin II attenuates the cardiac dysfunction induced by myocardial infarction. *Scientific Reports*.

[60] Pan Y., Zhou Z., Zhang H. (2019). The ATRQ*β*-001 vaccine improves cardiac function and prevents postinfarction cardiac remodeling in mice. *Hypertension Research*.

[61] Wakayama K., Shimamura M., Suzuki J.-I. (2017). Angiotensin II peptide vaccine protects ischemic brain through reducing oxidative stress. *Stroke*.

[62] Zhou Y., Wang S., Qiu Z. (2016). ATRQ*β*-001 vaccine prevents atherosclerosis in apolipoprotein E-null mice. *Journal of Hypertension*.

[63] Kutzler M. A., Weiner D. B. (2008). DNA vaccines: ready for prime time?. *Nature Reviews Genetics*.

[64] Koriyama H., Nakagami H., Nakagami F. (2015). Long-term reduction of high blood pressure by angiotensin II DNA vaccine in spontaneously hypertensive rats. *Hypertension*.

[65] Nochi T., Yuki Y., Takahashi H. (2010). Nanogel antigenic protein-delivery system for adjuvant-free intranasal vaccines. *Nature Materials*.

